# Report on the presence of a group of golden-headed lion tamarins (*Leontopithecus chrysomelas*), an endangered primate species in a rubber plantation in southern Bahia, Brazil

**DOI:** 10.5194/pb-4-61-2017

**Published:** 2017-03-14

**Authors:** Kristel M. De Vleeschouwer, Leonardo C. Oliveira

**Affiliations:** 1 Centre for Research and Conservation, Royal Zoological Society of Antwerp, 2018 Antwerp, Belgium; 2 Departamento de Ciências, Faculdade de Formação de Professores, UERJ, CEP 24435-005, São Gonçalo, RJ, Brazil; 3 Programa de pós-graduação em Ecologia e Conservação da Biodiversidade, Universidade Estadual de Santa Cruz, UESC, Salobrinho, CEP 45662-900, Ilhéus, BA, Brazil; 4 Bicho do Mato Instituto de Pesquisa, CEP 30360-082, Belo Horizonte, MG, Brazil

## Abstract

In a landscape fragmented by agriculture, the extent to which forest-dwelling primates can use the matrix between fragments can be critical for their long-term survival. So far, the golden-headed lion tamarin (*Leontopithecus chrysomelas*), an endangered primate inhabiting the Atlantic Forest of south Bahia, is only known to use shaded cacao (*Theobroma cacao*) agroforests within the matrix. We report on the use of a rubber plantation by a group of golden-headed lion tamarins between August 2013 and January 2014. The group used the rubber plantation on 16 of the 22 observation days (73 %), and we recorded behaviours such as eating, grooming and sleeping, consistent with the use of the area as a home range. We also observed associations with Wied's marmosets (*Callithrix kuhlii*). The locations of group sightings were not uniformly spread across the entire area of the rubber plantation, suggesting preferred use of certain areas. The presence of resources such as jackfruits (*Artocarpus heterophyllus*) and epiphytic bromeliads may be attracting both species to these plantations. In addition to shaded cacao plantations, rubber plantations with the appropriate structure may be a viable option for increasing forest connectivity for both species in south Bahia, reconciling economic rubber production with primate conservation.

## 1 Introduction

The conversion of forests for agriculture is a major cause of tropical forest fragmentation and degradation (Gibbs et al., 2010). The resulting landscape of smaller forest fragments embedded within a generally inhospitable matrix represents particular challenges to animals, especially rendering matrix-intolerant species more vulnerable to extinction (Benchimol and Peres, 2014). Matrix permeability critically determines dispersal between fragments, which in turn is essential for maintaining a sufficient level of gene flow and genetic diversity among small fragmented populations (Donald and Evans, 2006).

**Figure 1:**
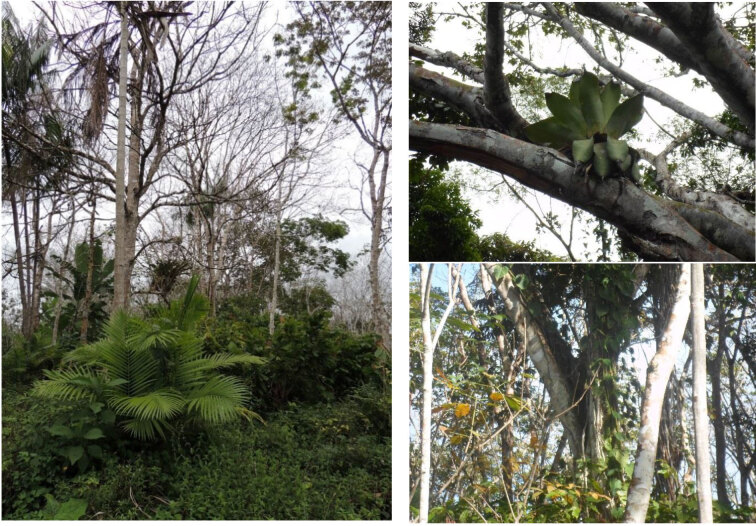
Representative images of the rubber plantation (Photos: Kristel De Vleeschouwer).

Forest-dwelling primates are particularly sensitive to the effects of anthropogenic forest fragmentation, with many species experiencing population decline or extinction (Benchimol and Peres, 2014). Loss of habitat due to agricultural expansion is the principal threat to primate populations worldwide, with livestock farming and ranching constituting the second most important threat in the Neotropics (Estrada et al., 2017). Yet high human population growth and poverty rates in primate-range countries, as well as global market economics, increase the likelihood of continued land-cover changes, forest loss and degradation (Estrada, 2013; Estrada et al., 2017). Documenting the ability of primates to use the matrix between fragments and identifying agricultural land-use practices compatible with primate survival, reproduction and/or dispersal across the landscape is imperative for developing guidelines for landscape management that reconcile primate conservation with subsistence of local communities (Estrada, 2013; Flesher, 2015).

Golden-headed lion tamarins (*Leontopithecus chrysomelas*) are frugivorous group-living primates of the Atlantic Forest of south Bahia (Brazil) that are classified as endangered and under threat from habitat loss, fragmentation and degradation (Kierulff et al., 2008). The landscape in south Bahia (Brazil) is a mosaic of Atlantic Forest remnants embedded within an agricultural matrix dominated by shaded cacao (*Theobroma cacao*) agroforests, locally known as *cabruca* (shaded cacao plantations in which the middle and understory trees of intact forests are removed and replaced with cacao trees; Alger and Caldas, 1994). Other economically important crops are present within the matrix, namely bananas (*Musa paradisiaca*) and rubber (*Hevea brasiliensis*), in addition to lesser quantities of coconut palm trees (*Cocos nucifera*) and manioc (*Manihot esculenta*) (IBGE, 2014). Golden-headed lion tamarin home ranges can encompass mature and degraded forests, in addition to *cabruca* (Raboy and Dietz, 2004). To date, no studies have reported the species using any other type of vegetation within the matrix, although the use of non-monoculture rubber plantations is suspected by former researchers in the area (B. E. Raboy, personal communication, 2014). Given that the long-term survival of this species may depend on its ability to use the matrix between fragments across its geographic range, this study aimed to investigate this potential use of rubber plantations.

We report on the presence of a group of golden-headed lion tamarins in a rubber plantation on a farm in south Bahia, thus constituting the first systematic observation of the species using this form of land use within the matrix; and we provide preliminary information on the group's behaviour and use of space in a fragmented landscape dominated by agricultural land use.

## 2 Methods

The fieldwork was conducted on a 29.4 ha privately owned farm, Fazenda Santo Antônio (-15.283796${}^{\circ }$; -39.134477${}^{\circ }$) in the municipality of Una (Bahia), in south Bahia. Approximately 35 % of the farm comprises patchy evergreen forest, while the remaining 65 % is planted with perennial rubber, cupuaçu (*Theobroma grandiflorum*), soursop (*Annona muricata*), pepper (*Piper nigrum*), bananas and ephemeral crops such as manioc and garden vegetables. The farm also includes a 30-year-old rubber plantation of approximately 2.5 ha surrounded by secondary forest patches, agricultural plantations and open fields. Many of the older rubber trees bear epiphytic bromeliads of various sizes. Additional trees, such as cacao, banana, jackfruit (*Artocarpus heterophyllus*) and guava (*Psidium guajava*), are interspersed with the rubber trees (Fig. 1).

**Table 1:** Data on total time spent with the focal group, use of natural vegetation types and rubber plantation, occurrence of associations with Wied's marmosets, use of sleeping sites and other behaviours observed over 22 observation days between August 2013 and January 2014.

Date	Total time with group (h:min)	Vegetation types used${}^{1}$	Seen in rubber plantation?	Seen in association?	Identity${}^{2}$ and location of sleeping tree			Behaviours observed while in rubber plantation
					Morning	Evening	Location	
12/08/2013	1:45	–	Yes	Yes	–	OJR01	Rubber plantation	Intraspecific and interspecific grooming, sleeping
19/08/2013	0:03	–	Yes	No	–	OJR01.	Rubber plantation	Sleeping
22/08/2013	2:30	MS	No	Yes	–	–		–
23/08/2013	3:19	MS	No	Yes	–	–		–
2/10/2013	3:05	MS	Yes	No	–	OJR01	Rubber plantation	Eating jackfruit, travelling, sleeping
3/10/2013	6:29	MS.	Yes	Yes	OJR01	–	Rubber plantation	Travelling, hiding, sleeping
22/10/2013	0:59	–	Yes	Yes	OJR01	–	Rubber plantation	Travelling, sleeping(together with marmosets)
23/10/2013	5:18	AS, IS	No	No	–	–		–
24/10/2013	4:03	AS, IS	Yes	Yes	OJR01	–	Rubber plantation	Eating jackfruit, travelling, sleeping
29/10/2013	6:30	–	Yes	Yes	OJR01	–	Rubber plantation	Travelling, sleeping
4/11/2013	2:07	MS	No	No	–	–		–
5/11/2013	5:41	–	Yes	Yes	OJR01	–	Rubber plantation	Travelling, sleeping
6/11/2013	5:20	AS	No	No	OJR02	–	AS	–
13/11/2013	6:07	AS	Yes	No	OJR01	–	Rubber plantation	Travelling, sleeping
14/11/2013	5:29	–	Yes	Yes	OJR01	–	Rubber plantation	Eating jackfruit, travelling, sleeping
15/11/2013	3:54	–	Yes	Yes	OJR01	–	Rubber plantation	Eating jackfruit, travelling, grooming, sleeping
20/11/2013	0:48	–	Yes	Yes	OJR01	–	Rubber plantation	Eating jackfruit, travelling, sleeping
4/12/2013	1:28	RF	Yes	No	OJR01	–	Rubber plantation	Eating jackfruit, travelling, sleeping
6/01/2014	1:00	RF	Yes	No	OJR01	–	Rubber plantation	Travelling, sleeping
14/01/2014	0:26	–	Yes	Yes	OJR01	–	Rubber plantation	Travelling, sleeping, scentmarking
21/01/2014	1:35	MS, AS	No	No	–	–		–
24/01/2014	1:21	–	Yes	No	OJR01	–	Rubber plantation	Travelling, sleeping

${}^{1}$ Abbreviations used: AS, advanced stage secondary vegetation; MS, medium stage secondary vegetation; IS, initial stage secondary vegetation; RF, regenerating field.${}^{2}$ Individual trees received unique codes composed of letters and a unique number.

**Figure 2:**
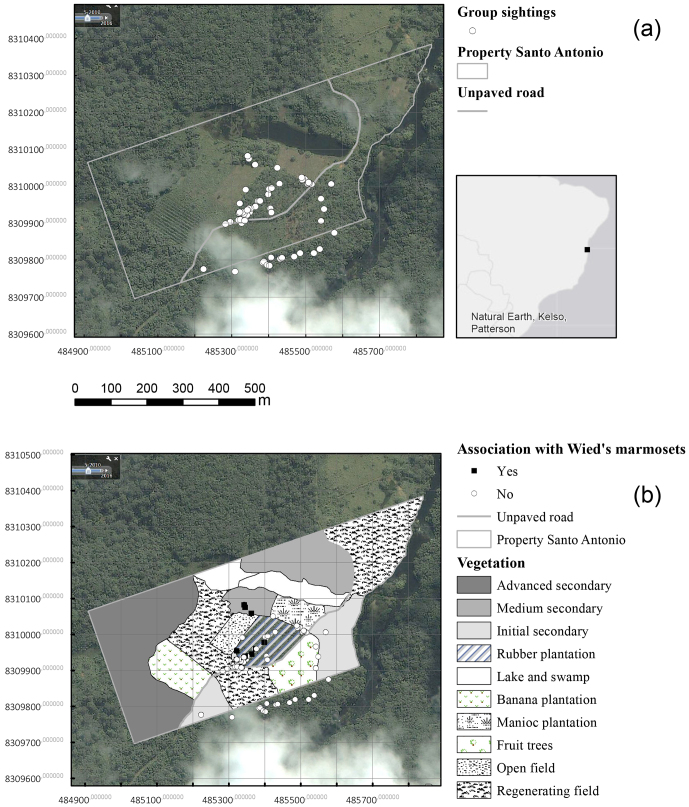
Maps of the study area, based on the interpretation of a georeferenced aerial photo obtained from Google Earth (image date: 27 May 2010). The upper map shows the property borders and locations where a group of golden-headed lion tamarins was sighted between August 2013 and January 2014. The map below provides information on the different natural vegetation types and forms of land use in different areas of the property, and the locations where the group was seen in association with Wied's marmosets. The black marker in the smallest frame indicates the location of the study area in Brazil.

### 2.1 Data collection

Following approval by the Brazilian authorities (Permit no. SISBIO N 23457-3), we employed a trained field assistant to start searching daily for groups of golden-headed lion tamarins on the farm. We recorded their behaviour using the following categories: travelling, remaining stationary, foraging for fruit, eating fruit, foraging for flowers, eating flowers, foraging for animals, eating animals, socialising, resting and “other”, as described in the ethogram in Raboy and Dietz (2004). From August 2013 through January 2014, we conducted ad libitum observations to identify groups of golden-headed lion tamarins. To facilitate habituation and monitoring, we captured a group of three golden-headed lion tamarins on 28 September 2013 in a degraded forest patch adjacent to the rubber plantation, using platforms armed with Tomahawk live traps (trap measures: 48.3 cm × 15.2 cm × 15.2 cm) and baited with bananas (following procedures described in Dietz et al., 1996). In the field laboratory, the animals were identified as two males and one female weighing 614, 617 and 637 g respectively, indicating adult ages (adult weight range: 500–800 g; Oliveira et al., 2011). One male and the female were fitted with a radio collar (Holohil, RI-2D) to facilitate ongoing habituation and monitoring. All procedures complied with legal requirements from Brazil and applicable international and institutional guidelines for the care and use of animals.

Subsequently, we started the habituation process. We located the group on 1–4 days/week at their sleeping site in the morning using radio-telemetry. We followed the group for as long as possible during the day until it retired to a sleeping site for the night. We terminated observations if we had the impression that our presence was causing the group to hide and might have prevented them from foraging sufficiently before locating a suitable sleeping tree for the night (see Table 1).

We recorded the following information at 20 min intervals: GPS location of the group (using a handheld GPS 92Xs unit); habitat type (rubber plantation); advanced, medium or initial secondary forest; open or regenerating fields (for definitions see Catenacci et al., 2009); and agricultural areas; in addition to ad libitum observations on behaviour. We did not calculate the total time the group spent in each vegetation type because of a possible bias as a result of the process of habituation.

Group locations were subsequently entered into ArcGIS 10.4.2 and plotted on a georeferenced image of the study area downloaded from Google Earth Pro (image date: 27 May 2010). We further added layers with additional information on the location of property borders as obtained from the owner's legal registration documents, and the location of different natural vegetation types and agricultural areas as identified on the ground.

## 3 Results

Between August 2013 and January 2014, we spent 103.5 h in the field, 69.28 h of which were in the presence of the group (meaning we either had visual contact or were within a few metres of the spot where we knew them to be hiding). We saw the group in the rubber plantation on 16 of 22 observation days (73 %) during which we were able to establish visual contact (Table 1; Fig. 2). Additional vegetation types used were initial, medium and advanced secondary forest, and a regenerating field.

On 17 occasions (77 % of observation days), we were able to determine the group's sleeping site in the morning or evening. In 16 of these 17 occasions (94 %), the group used one particular individual palm tree locally known as “catulé” (*Syagrus* sp., Family Arecaceae) at the northern edge of the rubber plantation, bordering a passion fruit plantation (Table 1; Fig. 2).

We saw Wied's marmosets (*Callithrix kuhlii*) together with the group in the rubber plantation on 10 of 16 sightings (63 %), occurring either in the rubber plantation or in the small patch of degraded forest located to the north of the rubber plantation (Table 1; Fig. 2). We observed the group of golden-headed lion tamarins using both this northern edge of the rubber plantation, where the palm tree sleeping site is located, and the area along a central line running north to south through the rubber plantation. We also observed the group in a patch of advanced secondary forest to the southeast of the rubber plantation encountering another group of golden-headed lion tamarins just across the border on the neighbouring property. We recorded the group eating in a jackfruit tree in the centre of the plantation. We additionally observed allogrooming, feeding and scent-marking behaviour during waking hours in the rubber plantation, in addition to hiding and travelling.

## 4 Discussion

This study is the first to provide evidence of the use of a rubber plantation in south Bahia by golden-headed lion tamarins. We observed rubber plantation use on the majority of study days. On several occasions, we saw the group in association with Wied's marmosets and observed behaviours such as eating, sleeping and grooming, thus suggesting that the group may have been using the plantation as part of its home range and not merely as a corridor between fragments. We further observed selective site use, in the sense that the group used some areas of the rubber plantation more than others; however, given the preliminary nature of our observations and the limited number of observation days (due to being early in the habituation process), further investigation is needed to confirm this pattern. We are currently following this and two more groups to study how they use rubber plantations spatially (entire area vs. edges; specific pathways) and to obtain detailed information on their resource use and behaviour.

Golden-headed lion tamarins have long been thought to depend on mature forests for survival, due to their requirement of resources such as epiphytic bromeliads for foraging and tree holes for sleeping (Rylands, 1996). More recent studies have reported the species being able to survive and reproduce in secondary forests, including *cabruca* agroforests (Catenacci et al., 2016; Oliveira et al., 2011). Being predominantly a frugivore, the species' diet varies between areas, reflecting differences in floristic composition and plant density; this indicates that the species adapts easily to changes in fruit availability (Catenacci et al., 2016). The extent to which the availability of sufficient sleeping sites and/or foraging sites limits its capacity for exploring altered habitats is as of yet undocumented. Therefore, maintaining a forest mosaic that includes patches of forest providing these resources is considered important (Catenacci et al., 2016). The rubber plantation in this study is relatively old (approx. 30 years) and infrequently managed through annual removal of undergrowth. With respect to potential resources for golden-headed lion tamarins, several of the older rubber trees bear epiphytic bromeliads, providing potential microhabitats for insect foraging (Raboy and Dietz, 2004). Bromeliads are listed as principal foraging sites at all study areas where golden-headed lion tamarins have been observed (Raboy and Dietz, 2004; Guidorizzi, 2008; Oliveira et al., 2011; Catenacci et al., 2016). Although we did not record our study group foraging in bromeliads in the rubber plantation during the observation period described here, we observed this behaviour at a later time. We suspect the lack of bromeliad foraging during the study period to be due to the habituation process, which frequently caused the group to hide, usually in bromeliads, and made the visualisation of feeding and foraging behaviour more difficult.

In addition to bromeliads, the rubber plantation contains some native fruit trees from plants included in the species' diet (e.g. *Tapirira guianensis*; Oliveira et al., 2010) and jackfruit trees. Jackfruit, an exotic species introduced in Brazil in the 18th century, is widely distributed along the Brazilian coast (Correia, 1975). Many animal species consume its fruits, including *Leontopithecus chrysomelas*; the consumption of jackfruit has been observed at all sites in the ombrophyllous forest where this primate has been studied (Raboy and Dietz, 2004; Oliveira et al., 2011; Catenacci et al., 2016).

All of these resources may attract golden-headed lion tamarins to these plantations and could therefore be used as a parameter to evaluate the suitability of rubber plantations for use by golden-headed lion tamarins and possibly Wied's marmosets. We advocate further investigation of this hypothesis, since the gained knowledge may help in defining management practices that aim at increasing the suitability of rubber plantations for both species.

### 4.1 Implications for conservation

Rubber agroforest systems in Asia can support local biodiversity and constitute an important component of sustainable land use, reconciling conservation with local community subsistence and development (Warren-Thomas et al., 2015). The present study demonstrates that such systems may also be used by golden-headed lion tamarins in the fragmented forests of south Bahia. Cacao is the main economic product of Bahia, particularly so in the municipalities within the species' distribution range (with 547 722 ha destined for production), followed by bananas. However, rubber constitutes the third-ranking crop in terms of area reserved for harvesting (IBGE, 2014), with 33 521 ha of land destined for rubber production in Bahia, approximately one third of which is located within the distribution range of the golden-headed lion tamarin (IBGE, 2014). In economic terms, rubber offers the additional benefit of guaranteeing a steady monthly income for farmers, since the trees provide a daily harvestable flow of latex in contrast to cacao which is harvested seasonally, thus providing an income during only a few months of the year (Schroth and Ruf, 2014). Rubber can be an important additional crop, particularly for farms too small to produce enough cacao to generate income for an entire year. In addition to shaded cacao plantations, rubber plantations with a structure that facilitates use by groups of golden-headed lion tamarins, either as a corridor or an extension of their home range, may constitute a land-use type that ensures functional connectivity between forest fragments for golden-headed lion tamarins, reconciling local development and conservation while adding value to rubber production and increasing the available habitat for these endangered primates.

## Data Availability

The data set on which this article was based on is available at (De Vleeschouwer and Oliveira, 2017).
